# Vaccination against SARS-CoV-2 in a rural Ugandan population

**DOI:** 10.1016/j.jvacx.2023.100355

**Published:** 2023-07-25

**Authors:** Joseph Mugisha, Bernard Mpairwe, Beatrice Kimono, Pontiano Kaleebu, Robert Newton

**Affiliations:** MRC/UVRI & LSHTM Uganda Research Unit, Entebbe, Uganda

## Abstract

Working within the context of a longstanding cohort in rural southwestern Uganda (the General Population Cohort), we collect health-related data in successive survey rounds from all residents of 25 adjacent villages on a biannual basis. Between January 2022 and July 2022, 2318 adult participants in the cohort were asked about their SARS-CoV-2 vaccination status; 80% of participants had received at least one dose of vaccine and 51% had received two doses; 2% had received a third dose.

## Dear Editor

Data on vaccination rates against SARS-CoV-2 in sub-Saharan Africa are relatively sparse and reporting usually relates to the number of doses delivered, rather than to the number of people vaccinated. Since dosing schedules for SARS-C0V-2 require a minimum of two doses (with the exception of the Johnson & Johnson single-dose vaccine, which was used extensively on the continent), it is unclear what proportion of the population are fully vaccinated. In Uganda, between March 2021 and July 2022, more than 24 million doses have been delivered, enough to fully vaccinate about 30% of the adult population (18+ years).

Working within the context of a longstanding cohort in rural southwestern Uganda (the General Population Cohort) [1], we collect health-related data in successive survey rounds from all residents of 25 adjacent villages on a biannual basis. Between January 2022 and July 2022, 2318 adult participants in the cohort were asked about their SARS-CoV-2 vaccination status; 53% were women and the age distribution reflects that of the background population. Data are shown in Table 1: 80% of participants had received at least one dose of vaccine and 51% had received two doses; 2% had received a third dose. Prevalence of vaccination was slightly lower among the youngest and the oldest and, was higher among women than men, but the differences were modest. No information was available on which vaccine was delivered to the participants.

**Table 1:** Prevalence of SARS-CoV-2 in a rural Ugandan population.

	**Category**	**Percent (number/total)**
Vaccinated with ≥ 1 dose	Yes	80% (1855/2318)
	No	20% 463/2318)
Doses received among vaccinated	One	47% (879/1855)
	Two	51% (947/1855)
	Three	2% (29/1855)
Age vaccinated (89 missing values) with ≥ 1 dose	18–30	73% (501/683)
	31–40	80% (378/471)
	41–50	84% (362/430)
	51–60	84% (281/334)
	61–70	87% (164/188)
	71+	75% (92/123)
Sex of vaccinated	Male	75% (638/857)
	Female	83% (1217/1461)

For unvaccinated individuals, data were also collected on the reasons why: 52% refused vaccine for fear of side effects; 11% claimed that they lived too far from a health facility; 10% did not perceive that they were at risk for COVID-19 and 27% refused vaccine for reasons of sickness, pregnancy or they were too busy. There remain significant numbers of people in this rural Ugandan population who are either not vaccinated at all, or who are only partially vaccinated.

**Funding**

The work was conducted at the MRC/UVRI & LSHTM Uganda Research Unit which is jointly funded by the UK Medical Research Council (MRC), part of UK Research and Innovation (UKRI) and the UK Foreign, Commonwealth and Development Office (FCDO) under the MRC/FCDO Concordat agreement and, is also part of the EDCTP2 programme supported by the European Union.

## Declaration of Competing Interest

The authors declare that they have no known competing financial interests or personal relationships that could have appeared to influence the work reported in this paper.

## Data Availability

Data will be made available on request.
